# Thyroid metastasis from cervical carcinoma

**DOI:** 10.1016/j.bjorl.2023.03.005

**Published:** 2023-03-22

**Authors:** Shuto Hayashi, Takumi Kumai, Tomohiko Michizuka, Takashi Osaki

**Affiliations:** a Nikko Kinen Hospital, Department of Otorhinolaryngology, Hokkaido, Japan; b Asahikawa Medical University, Department of Otolaryngology-Head and Neck Surgery, Hokkaido, Japan; c Asahikawa Medical University, Department of Innovative Head & Neck Cancer Research and Treatment (IHNCRT), Hokkaido, Japan

## Introduction

Metastases to the thyroid gland have been reported in 1.4%–3% of patients who have undergone thyroidectomy for malignancy^1^. The most common sites of primary tumors that metastasize to the thyroid are the kidneys (25%), lungs (22%), gastrointestinal tract (13%), and breast (13%)^2^. Cervical carcinomas frequently metastasize to the lungs (21%), bone (16%), para-aortic lymph nodes (11%), abdominal cavity (8%), and supraclavicular lymph nodes (7%); however, primary gynecological malignancies rarely metastasize to the thyroid gland. This article describes a rare case of cervical squamous cell carcinoma that metastasized to the thyroid gland.

## Case presentation

A 69-year-old woman presented to our department with a 2-month history of progressive swelling in the right anterior neck. Sixteen years ago, the patient had undergone a left hemithyroidectomy for a benign thyroid tumor at another hospital, which resulted in persistent paralysis of the left recurrent laryngeal nerve. Further, she had undergone hysterectomy followed by chemoradiotherapy for cervical squamous cell carcinoma 5 years before admission. The patient refused further examination and treatment for the cervical cancer.

**Figure 1:**
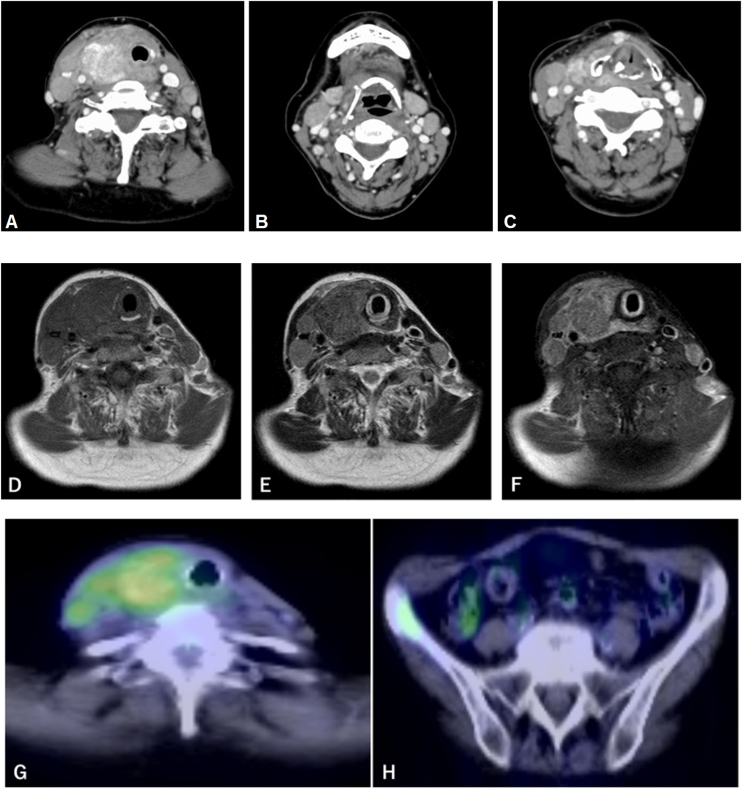
CT, MRI, and FDG-PET/CT images. (A–C) Contrast-enhanced Computed Tomography (CT) image showing a shadow with irregular margins and calcification in the right thyroid gland and swollen bilateral lymph nodes. (D‒F) Magnetic Resonance Imaging (MRI) showing a right thyroid tumor with low signal intensity on T1-weighted images (D), iso-signal intensity on T2-weighted images (E), and heterogeneous strong enhancement by gadolinium (F). (G‒H) Fluorodeoxyglucose Positron Emission Tomography/CT (FDG-PET/CT) image showing abnormal FDG accumulation in the right thyroid gland, bilateral cervical lymph nodes, and right iliac bone.

Physical examination revealed a hard nodule with a diameter of 5 cm in the right anterior cervical lesion. A laryngeal fiberscope revealed paralysis of the left, but not right larynx. Contrast-enhanced Computed Tomography (CT) revealed an irregular calcified tumor in the right thyroid gland and swollen lymph nodes in the bilateral neck (Fig. 1 A–C). Magnetic resonance imaging revealed a right thyroid tumor with low signal intensity on T1-weighted images and iso-signal intensity on T2-weighted images; moreover, the tumor showed heterogeneous staining with gadolinium (Fig. 1 D–F). Fluorodeoxyglucose Positron Emission Tomography/CT (FDG-PET/CT) showed abnormal FDG accumulation in the right thyroid gland, bilateral neck lymph nodes, and right iliac bone (Fig. 1 G–H). Fine Needle Aspiration Cytology (FNAC) of the thyroid revealed atypical epithelial cells with a high nuclear/cell ratio and hyperchromatic nuclei, which resembled cervical cancer cells.

**Figure 2:**
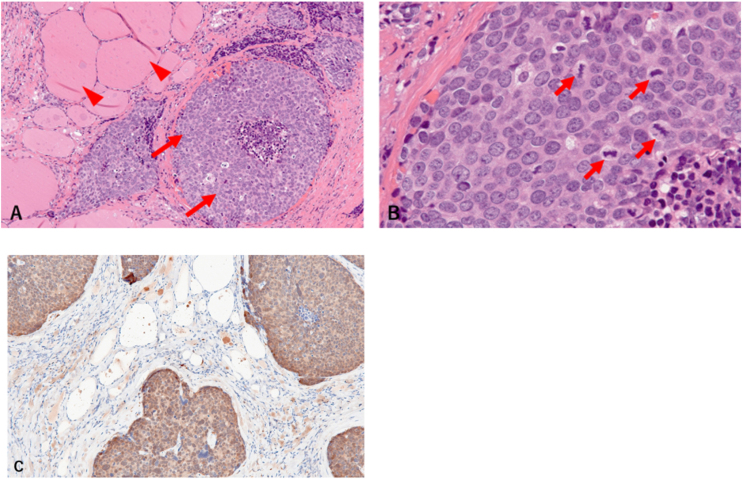
Pathological findings. (A) Normal thyroid tissue (arrowhead) with atypical epithelial cell proliferation (arrow). (B) Atypical cells with oval nuclei, uneven chromatin aggregation, and multiple fissions (arrowheads). (C) Atypical cells expressed p16.

Given the risk of airway obstruction and dysphagia, the patient underwent tracheostomy and right hemithyroidectomy. A right neck dissection was simultaneously performed due to adherence of the thyroid tumor to the cervical lymph nodes. Under general anesthesia, a J-shaped skin incision was made from the right mastoid process to the anterior neck. The sternocleidomastoid muscle and internal jugular veins were resected due to tumor invasion. The thyroid tumor invaded the trachea and esophagus, which were both preserved following their surface resection. Pathological examination revealed normal thyroid tissue with squamous cell carcinoma (Fig. 2 A–B). Since the atypical epithelial cells were positive for p16, which is a surrogate marker of Human Papillomavirus (HPV), the patient was diagnosed with thyroid metastasis from cervical cancer (Fig. 2C). The patient has remained alive without any symptom for 5 postoperative months.

## Discussion

Cervical cancer is related to HPV and expresses p16 as a surrogate marker for HPV infection. Our patient had a history of cervical cancer and did not present with other HPV-related cancers, including oropharyngeal cancer. Since histological examination revealed squamous cell carcinoma with p16 staining, the patient was diagnosed with thyroid metastasis from cervical cancer.

Metastasis of cervical cancer to the thyroid gland is rare, with only 14 cases having been reported, including our case (Table 1)^3^, ^4^, ^5^, ^6^, ^7^, ^8^, ^9^, ^10^, ^11^, ^12^, ^13^, ^14^. The mean age of the reported patients was 55 years (range: 36–72 years; *n* = 14). The histology of cervical cancer was squamous cell carcinoma in nine cases, adenocarcinoma in two cases, neuroendocrine carcinoma in two cases, and poorly differentiated carcinoma in one case. Moreover, the laterality of the thyroid tumor was bilateral, right, and left in one, six, and four patients, respectively. The median latency between the initial diagnosis of cervical cancer and its metastases to the thyroid gland was 15 months (range: 5–12 years; *n* = 12). FNAC was performed in nine cases; among them, seven cases were considered positive for malignant cells. Thyroidectomy was performed in seven patients to preserve their quality of life. Distant metastasis other than thyroid tumors occurred in 10 out of the 14 patients (71%); among them, eight patients died within a year due to multiorgan metastasis.

**Table 1:** Reported cases of metastatic cervical carcinoma to the thyroid gland.

Case	Year	Author	Age	Histology	Side	Distant metastasis	Latency period	FNAC	Treatment	Progress
1	1977	Martino et al.^4^	39	SCC	Lt	Lung	2 years	NS	PCO	DOD (4 months)
2	2000	Cheyng et al.^5^	57	AC	Bil	None	1 year	NS	TT + RT	NS
3	2002	Singh et al.^6^	38	NC	Rt	Liver, lung	1 year	SOM	CT	DOD (6 months)
4	2005	Kim et al.^7^	42	SCC	NS	None	6 years	SOM	PCO	DOD (4 months)
5	〃	〃	53	SCC	NS	Pancreas	1.5 years	ND	PCO	DOD (6 months)
6	2006	Karapanagiotou et al.^8^	68	SCC	Rt	Lung	4 years	ND	CT + RT	DOD (4 months)
7	2013	Vamsy et al.^9^	68	SCC	Lt	Bone, liver, lung	12 years	SOM	TT + RND + CRT	NS
8	2015	Fuentes-Martinez et al.^10^	36	PDC	Rt	Kidney	1 year	SOM	CRT	DOD (6 months)
9	2016	Celik et al.^11^	56	SCC	Rt	Bone, lung	6 months	NS	TT + CNLND	DOD (5 months)
10	2019	Varli et al.^12^	55	SCC	NS	None	5 months	NS	TT + CT + RT	NS
11	2021	Bertone F et al.^13^	72	AC	Rt	None	NS	SOM	HT + CT	AWD (6 months)
12	2021	Ravindrakumar et al.^14^	56	SCC	Lt	Bone	NS	SOM	CRT	NS
13	2022	Li et al.[15]	54	NC	Lt	Bone	3 years	NS	HT + CNLND + CT	DOD (1 year)
14	2022	Present case	69	SCC	Rt	Bone	5 years	SOM	HT + RND	AWD (5 months)

AC, Adenocarcinoma; AWD, Alive With Disease; Bil, Bilateral; CNLND, Central Neck Lymph Node Dissection; CRT, Chemoradiotherapy; CT, Chemotherapy; DOD, Died of Disease; HT, Hemithyroidectomy; Lt, Left; NC, Neuroendocrine Carcinoma; ND, No Diagnosis; NS, Not Stated; PCO, Palliative Care Only; PDC, Poorly Differentiated Carcinoma; RND, Radical Neck Dissection; Rt, Right; RT, Radiotherapy; SCC, Squamous Cell Carcinoma; SOM, Suspicion Of Metastasis; TT, Total Thyroidectomy.

Since thyroid tumors can obstruct the airway and digestive tract, it is important to carefully treat metastasis to the head and neck region. Since there are no international guidelines for the management of thyroid metastases from cervical cancer, individualized treatment interventions including tracheostomy and surgical resection should be considered to relieve symptoms and improve quality of life. Since our patient had left recurrent laryngeal nerve paralysis and thyroid tumor invasion near the right recurrent laryngeal nerve, a tracheostomy was performed to secure the airway. Additionally, thyroidectomy and neck dissection were performed to preserve swallowing function by releasing the esophagus from the adhered tumor. Although these surgeries are not curative treatments for cervical cancer with multiple metastases, palliative tumor reduction could improve the quality of life.

## Conclusion

This article reports a rare case of thyroid metastasis from cervical carcinoma. Although the prognosis of cervical cancer with metastasis is generally poor, surgical resection may be useful for securing the airway and digestive tract in order to temporarily improve the quality of life of patients with metastatic thyroid cancer.

## Funding

The authors received no financial support for the publication of this article.

## Conflicts of interest

The authors declare no conflicts of interest.
